# Editorial: Insights in lipids in cardiovascular disease: 2021

**DOI:** 10.3389/fcvm.2022.940153

**Published:** 2022-07-15

**Authors:** Irena Levitan, Tomas Vaisar

**Affiliations:** ^1^Department of Medicine, Pulmanary Section, University of Illinois at Chicago, Chicago, IL, United States; ^2^Department of Medicine, UW Medicine Diabetes Institute, University of Washington, Seattle, WA, United States

**Keywords:** LDL, VLDL, HDL, triglycerides, lipidomics

The connection between lipids and cardiovascular disease emerged almost a 100 years ago when it was recognized that familial hypercholesterolemia, a genetic disorder that results in high levels of low-density lipoproteins (LDL) leads to a high risk of atherosclerosis. Since then large-scale clinical and epidemiological studies have revealed a direct association between LDL and cardiovascular disease (CVD), whereas high-density lipoproteins (HDL) were found to be negatively correlated. The vast majority of studies that have investigated the effects of LDL and HDL have primarily focused on their cholesterol content. In this Research Topic, we included several articles that focus on LDL and HDL heterogeneities, as well as on triglyceride rich lipoproteins, from perspectives other than their cholesterol content and their less well-studied effects.

## Not all LDLs are created equal

It is well recognized that there is significant heterogeneity in the composition and proatherogenic properties of LDL particles with small, dense LDL (sdLDL) being more proatherogenic than larger particles. A review article by Jin et al. on “*Small, Dense Low-Density Lipoprotein-Cholesterol and Atherosclerosis: Relationship and Therapeutic Strategies*” provides an in-depth analysis of the properties, mechanisms of action, and methods of detection of sdLDL, and describes therapeutic interventions. One of the main conclusions is that the small size and higher density of sdLDL particles enhance their ability to penetrate the vascular wall affecting lipid metabolism and promoting inflammation. The molecular mechanisms specific to sdLDL-induced proatherogenic effects are not well understood and further studies are needed to address these questions.

## HDL: Quality, not quantity

Another staple of lipoprotein research that emerged over the past decade is the complexity of HDL. Despite epidemiological evidence for inverse association of HDL cholesterol content (HDL-C) with CVD, genetic studies and therapeutic interventions elevating HDL-C do not support the causal role of HDL-C in CVD. A meta-analysis of Lee et al. “*Cholesterol Efflux Capacity and Its Association With Adverse Cardiovascular Events: A Systematic Review and Meta-Analysis”* provides a new perspective highlighting the capacity of HDL to mediate cholesterol efflux rather than HDL-C concentration. Analyzing 20 trials, the authors show that an increase in cholesterol efflux capacity (HDL-CEC) is associated with improved outcomes even when adjusted for the levels of HDL-C. This analysis shows that HDL-CEC rather than HDL-C concentration should be taken into the account both for clinical applications and for basic research.

A review by Diab et al. “*HDL Composition, Heart Failure, and Its Comorbidities”* comprehensively summarizes the state of the knowledge of the relationship between HDL composition and function and heart failure, both from the perspective of known HF comorbidities (inflammation, diabetes, obesity, and renal disease) on HDL and from the perspective of altered HDL functions: including anti-inflammatory, anti-oxidative and anti-fibrotic. In particular, the role of apoA-I/SRBI, apoM, and sphingosine-1-phosphate is emphasized in relation to endothelial and vascular protection. Notably, the discussion explores the positive results of some HDL targeted therapeutics that showed promising results in relation to HF.

These two articles highlight that HDL should not be completely written off after the failures of the CETP inhibitor trials and emphasize the promising areas where HDL could play an important protective role.

## Beyond LDL

It is also increasingly recognized that multiple lipids, beyond the major lipoprotein species, play a major role in CVD and more studies are needed to elucidate these effects. In this Research Topic, we present several studies that explore the lipidomics of cardiovascular disease.

Triglycerides (TG), esters of glycerol and free fatty acids, are a major component of larger lipoproteins [triglyceride rich lipoproteins (TRL)], especially very low-density lipoproteins (VLDL), and are associated with increased CVD, even in people with well controlled LDL-C. However, the mechanisms of TRL-induced detrimental effects are not well understood. The study by Lin et al. on “*Dietary-Induced Elevations of Triglyceride-Rich Lipoproteins Promote Atherosclerosis in the Low-Density Lipoprotein Receptor Knockout Syrian Golden Hamster*” develops a new animal model to study the effects of TRL and discriminate between the effects of TRL and LDL. The authors show that blocking intestinal uptake of cholesterol results in a decrease in the TRL levels but not LDL and that this is sufficient to reduce atherosclerosis. This model is a powerful new tool to elucidate the mechanisms by which triglyceride rich lipoproteins contribute to CVD.

One of the apolipoproteins that emerged as a regulator of TRLs and a potential therapeutic target is apolipoprotein C-III (APOC3) through its effects on LPL activity and liver lipoprotein uptake. Olivieri et al. “*High Plasma Concentration of Apolipoprotein C-III Confers an Increased Risk of Cerebral Ischemic Events on Cardiovascular Patients Anticoagulated With Warfarin”* explores yet another role of the APOC3 as pro-coagulant in people on warfarin and its association with increased risk of cerebral ischemic events. The study shows that people with high APOC3 had a 3x higher risk of stroke or transient ischemic attack than those with low APOC3. Although the specific molecular mechanism whether due to the TRL elevating effect of APOC3 or APOC3s pro-coagulation activity remains to be elucidated, the study highlights yet another way by which APOC3 contributes to cardiovascular disease.

An underappreciated subfraction of TRLs, is intermediate density lipoprotein (IDL), a class of lipoprotein that encompasses at least some portion of remnant lipoproteins (RLP), lipoproteins formed by lipolysis of VLDL and chylomicrons. While RLPs are not easy to measure, the measurement of IDL may serve as their surrogate marker. In their study “*Clinical Significance of Intermediate-Density Lipoprotein Cholesterol Determination as a Predictor for Coronary Heart Disease Risk in Middle-Aged Men*” Yoshida et al., quantify IDL-C using anion-exchange chromatography to demonstrate a significant association of IDL-C with an increased 10-year risk of CVD as estimated by Framingham risk score (FRS). Notably, in a step-wise multivariate logistic analysis, the IDL-C was a stronger predictor of the higher FRS than VLDL-C or other traditional risk factors.

Collectively, these studies demonstrate that targeting LDL-C as the only lipoprotein therapeutic target is not sufficient and that both smaller (HDL) and larger (TRLs and especially RLP and/or IDL) lipoproteins play important roles in residual cardiovascular risk after controlling LDL-C.

## Author contributions

All authors listed have made a substantial, direct, and intellectual contribution to the work and approved it for publication.

## Funding

This research was supported by NIH NHLBI grants R01HL141120, R01HL083298, and R01HL122010 to IL and R01HL144558, P01HL151328, and P01HL128203 to TV.

## Conflict of interest

The authors declare that the research was conducted in the absence of any commercial or financial relationships that could be construed as a potential conflict of interest.

## Publisher's note

All claims expressed in this article are solely those of the authors and do not necessarily represent those of their affiliated organizations, or those of the publisher, the editors and the reviewers. Any product that may be evaluated in this article, or claim that may be made by its manufacturer, is not guaranteed or endorsed by the publisher.

